# Removal of steroid estrogens from municipal wastewater in a pilot scale expanded granular sludge blanket reactor and anaerobic membrane bioreactor

**DOI:** 10.1080/09593330.2015.1070922

**Published:** 2015-09-07

**Authors:** Ayumi Ito, Lawson Mensah, Elise Cartmell, John N. Lester

**Affiliations:** ^a^ Cranfield Water Science Institute, School of Energy, Environmental Technology and Agrifood, Cranfield University, BedfordshireMK43 0AL, UK; ^b^ Department of Civil and Environmental Engineering, Faculty of Engineering, Iwate University, MoriokaIwate 020–8550, Japan

**Keywords:** anaerobic treatment, municipal wastewater, fortification, temperate, steroid estrogens

## Abstract

Anaerobic treatment of municipal wastewater offers the prospect of a new paradigm by reducing aeration costs and minimizing sludge production. It has been successfully applied in warm climates, but does not always achieve the desired outcomes in temperate climates at the biochemical oxygen demand (BOD) values of municipal crude wastewater. Recently the concept of ‘fortification' has been proposed to increase organic strength and has been demonstrated at the laboratory and pilot scale treating municipal wastewater at temperatures of 10–17°C. The process treats a proportion of the flow anaerobically by combining it with primary sludge from the residual flow and then polishing it to a high effluent standard aerobically. Energy consumption is reduced as is sludge production. However, no new treatment process is viable if it only addresses the problems of traditional pollutants (suspended solids – SS, BOD, nitrogen – N and phosphorus – P); it must also treat hazardous substances. This study compared three potential municipal anaerobic treatment regimes, crude wastewater in an expanded granular sludge blanket (EGSB) reactor, fortified crude wastewater in an EGSB and crude wastewater in an anaerobic membrane bioreactor. The benefits of fortification were demonstrated for the removal of SS, BOD, N and P. These three systems were further challenged with the removal of steroid estrogens at environmental concentrations from natural indigenous sources. All three systems removed these compounds to a significant degree, confirming that estrogen removal is not restricted to highly aerobic autotrophs, or aerobic heterotrophs, but is also a faculty of anaerobic bacteria.

## Introduction

In the European Union and most developed countries demands on wastewater effluent quality have been increasing.[1,2] The original benchmark standard of the Royal Commission 1898–1915 [3] of 30:20 (30 mg l^−1^, suspended solids (SS) 20 mg l^−1^ Biochemical Oxygen Demand (BOD), diluted 8:1 with eight volumes of river water with a BOD of 2 mg l^−1^ or lower) has long been superseded. Water reuse led to 10:10 and 5:5 standards (Thames Conservancy, UK) in the 1960s.[4] Concern about nutrients nitrogen (N) and phosphorus (P) and their impact on surface water quality and in particular eutrophication led to denitrification and phosphorus removal technologies being developed [5,6] and widely implemented to achieve standards for N and P.[7]

The development of more stringent standards for wastewater effluents did not stop with SS, BOD, N and P and by the 1970s had embraced trace substances, originally referred to as the so-called ‘heavy metals' and organic micropollutants, most notably ‘drins' (organochlorine pesticides), Polychlorinated Biphenyls (PCBs – dielectric fluids) and Polynuclear Aromatic Hydrocarbons (PAHs – combustion products). These substances have been the subject of extensive studies.[8–10] Early research of these substances was divided between the totally conservative trace elements (heavy metals) and the organic micropollutants which exhibit three types of generalized behaviour, non-degradable (conservative), partially degradable (semi-conservative) and readily degradable (usually biodegradable).[8,11,12] Over the last 30 years a whole range of chemicals have assumed environmental importance; these include organometallics such as tributlytin, methylmercury and organo leads,[13–15] endocrine disruptors such as estrogens,[16–22] biocides,[23–25] pharmaceuticals [26–31] and Polybrominated Flame Retardants.[32,33] These substances are now collectively often called hazardous substances; this definition ignores chemical structure, environmental behaviour, degradability and uses only environmental hazard or risk as a definition for concern.[9]

Sewage sludge treatment and reuse were soon perceived as a major issue for the Water Industry in Europe, which was not helped by the cessation of marine disposal and increasingly stringent standards for application to Agricultural Land.[34] This problem was further exacerbated by increasingly stringent effluent standards which generated more sludge, that is, denitrification and phosphorus removal,[7] thus increasing the volume and the cost of sludge to be treated and reused. The cost of aeration for activated sludge treatment and the cost of sludge treatment and reuse are the two largest components of the operational expenditure for a wastewater treatment facility.[35,36] Both these costs could be dramatically reduced if anaerobic treatment was used for the treatment of municipal wastewater as no air is required and sludge yield is much lower.[35] Although this has been successfully implemented in tropical climates [37,38] the lower temperature in temperate zones coupled with the low substrate concentration of sewage has limited this type of treatment. Recently the concept of fortification has been proposed to overcome this problem [35] and has achieved promising results at the pilot scale.[39–41] The concept depends on only treating a portion of the flow (typically in the region of 50%) anaerobically using an expanded granular sludge blanket (EGSB) reactor. That portion of the crude sewage is fortified with the primary sludge from the primary settlement of the remaining crude sewage flow. This fortified sewage can be treated at temperatures between 10 and 17°C with good BOD removal and limited biomass production.[39–41] The effluent from the EGSB is combined with the settled sewage and then can subsequently be treated aerobically to produce a fully nitrified and denitrified effluent at a much lower cost than activated sludge alone and with phosphorus removal if required.

Combined anaerobic aerobic treatment offers several advantages over aerobic treatment alone. Some 50% of the crude wastewater passes directly to anaerobic treatment, without primary sedimentation. Thus the hydraulic retention time (HRT) of primary sedimentation, through which the remainder of the crude sewage passes, can be increased with concomitant increases in removals of total suspended solids (TSS) and BOD, or some primary sedimentation tanks can be decommissioned. Sludge production overall can be reduced by >90%, and energy savings of up to 67% are potentially achievable [35] compared with activated sludge treatment only.

When new treatment technologies are proposed for the treatment of wastewater, they must not only deal with the traditional pollutants for which the pre-existing technologies were originally put in place, for example BOD, SS, *N* and *P*, but also hazardous substances, at less overall economic and environmental costs than other strategies.

The study reported here was undertaken not only to establish if anaerobic aerobic treatment could remove BOD, SS, *N* and *P* with reductions in sludge production and oxygen consumption and consequently economic and environmental benefits, but also to establish the removal effectiveness for hazardous substances. For this purpose, estrogens were selected as a model class of such compounds.

## Material and methods

### The anaerobic experimental reactors

The pilot scale anaerobic reactors utilized in this study were an EGSB and an anaerobic membrane bioreactor (anMBR). The reactors were utilized as a side by side trial treating the same crude wastewater (supplied from a wastewater treatment works of 3000 population equivalent – PE, South East England, UK). The EGSB comprised a 0.19 m diameter × 1.5 m height Perspex column and was fitted with a lamella plate clarifier for solid/liquid/gas separation and resulted in a volume of 42.5 l (Paques, Balk, The Netherlands). When treating crude wastewater, an HRT and organic loading rate (OLR) of 4.8 and 9.6 h and *c.* 2–2.2 kg COD m^−3^ d^−1^, respectively, were used (COD: chemical oxygen demand), and an upflow velocity of *c.* 1.2 m h^−1^ was maintained by internal circulation which provided a granular bed expansion of *c.* 18%. The granular sludge inoculum (25 l) was sourced from a pulp mill effluent and was left to acclimatize for >3 months prior to experimentation. The anMBR comprised a 1600 l reactor volume and was fitted with a 12.5 m^2^ hollow fibre membrane with a nominal pore size of 0.085 µm. The OLR achieved in the anMBR was restricted by the operating flux of 6 l m^−2^ h^−1^ which set the HRT and OLR at 15.8 h and 0.5 kg COD m^−3^ d^−1^. The anMBR was operated for 20.8 months prior to analysis at which time the flocculated biomass had stabilized at 7.7 g l^−1^. The reactors were operated in parallel at temperatures in the range of 10–17°C. Sludge was not wasted from the reactors over the experimental period; only sludge samples were removed for analysis, which were deemed to be of negligible volume.

When treating wastewater fortified with primary sludge to increase the organic strength to the EGSB reactor, primary sludge (derived from the 3000 PE works) was blended into the EGSB wastewater feed. Prior to blending, the primary sludge (5.7% total solids (TS), total COD (tCOD) 71 g l^−1^) was sifted through a 10 mm metal mesh screen to remove gross solids and exposed to acoustic hydrolysis (ultrasound) to maximize the available soluble carbon substrate. Whilst not the preferred pre-treatment step due to the high specific energy input, ultrasound was an expedient experimental technique and was thus adopted for simplicity. The ultrasound unit comprised a 2 l flow cell and delivered a maximum power of 1 kW at 16–20 kHz. Sludge was passed through the cell for ca. 7.5  min, achieving an energy density of ca. 100 kJ l^−1^ and an increase in soluble chemical oxygen demand of 250%. During treatment of the fortified wastewater, the HRT was increased to 19.4 h which resulted in an OLR of 2.2 kgCOD m^−3^ d^−1^. The influent and effluent physicochemical characteristics and treatment performance are reported in Table 1.

**Table 1.** Operating conditions and treatment performance (average values) of the anaerobic EGSB reactor and MBR pilot scale reactor.

	EGSB		anMBR	
	Fortified (0.8 days HRT)	Unfortified (0.2 days HRT)	Unfortified (0.4 days HRT)	(0.7 days HRT)
Reactor volume (l)	43			1200
Flow rate (l d^−1^)	52.4	216	108	1800
HRT (h)	19.4	4.8	9.6	15.8
BOD (mg l^−1^) influent	1303	247	215	178
BOD (mg l^−1^) effluent	607	94	69	13
BOD removal (%)	55	61	66	91
tCOD (mg l^−1^) influent	1790	414	351	336
tCOD (mg l^−1^) effluent	769	161	127	74
tCOD influent load (kg m^−3^ d^−1^)	2.2	2.1	0.9	0.5
tCOD removal (%)	51	58	62	78
TSS (mg l^−1^) crude influent	1431	144	125	127
TSS (mg l^−1^) effluent	279	44	27	–
TSS removal (%)	80	65	78	–

### Analytical reagents and methodology for the analysis of steroid estrogens

Estrogen standards (>98% purity); oestrone (E1), 17β-estradiol, (E2), estriol (E3) and oestrone sulphate (E1–3S) were purchased from Sigma Aldrich (Dorset, UK). Deuterated internal standards; E1-d_4_, E2-d_5_, E3-d_3_, EE2-d_4_ and E1–3S-d_4_ sulphate were obtained from QMX Laboratories (Thaxted, UK). The high performance liquid chromatography grade solvents, acetone, methanol, dichloromethane, ethylacetate and hexane were purchased from Rathburn Chemicals (Walkerburn, UK). Ammonium hydroxide was ACS grade and obtained from Sigma Aldrich (Dorset, UK) and ultra-pure water of 18.2 MΩ quality (Elga, Marlow, UK) was used in the preparation of mobile phases. COD, ammoniacal nitrogen, nitrate, nitrite and total nitrogen (TN) proprietary cell test kits were purchased from VWR International (Leicestershire, UK).

For all samples collected aqueous and particulate phases were separated by centrifugation and filtration and analysed using the method based on Koh et al. [42] and further developed by Petrie et al.[26] The separated aqueous and particulate phases were then analysed separately using a three-stage extraction and clean-up procedure. Quantification was by ultra-performance liquid chromatography tandem mass spectrometry as previously described.[26] A triple quadrupole was utilized and two multiple reaction monitoring transitions were monitored per compound. Deuterated surrogates were used to compensate for any loss of analytes during sample preparation. The method achieved aqueous and particulate method detection limits of 5.0 × 10^−5^ to 1.7 × 10^−4^ µg l^−1^ and 2.5 × 10^−3^ to 8.9 × 10^−3 ^µg g^−1^, respectively. Steroid estrogen recoveries ranged from 46.9 to 114.3% for the aqueous and particulate phases.

### Analysis of routine parameters

The total and soluble COD, total phosphorus and TN were determined using Merck Spectroquant cell tests (Darmstadt, Denmark) according to the manufacturer's instructions. The BOD, TS, SS and volatile solid content and alkalinity were determined using standard methods [43] and the percentage methane in the biogas was determined by a gas analyser (Servomex 1440C, Crowborough, UK).

## Results and discussion

### Concentrations of estrogens in crude sewage

The concentration of E1 in the influent crude sewage when operating at 4.8 h HRT varied from 7.5 to 65 ng 1^−1^, for E2 from 0.8 to 14 ng l^−1^, for E3 from 45 to 68 ng 1^−1^ and for E1–3S from 30 to 43 ng 1^−1^ (Table 2). These values are typical for this site and others [44] which serve a small population (PE 3000) with a Dry Weather Flow of 650 m^3 ^d^−1^ and industrial inputs of <10%, these being from a local airfield. The site is characterized by a short retention time in the sewer system of <3 h. The latter is thought to be responsible for the high influent concentrations of E1–3S as deconjugation is highly time dependent.[45,46] It is evident from a comparison of the figures above for crude sewage with the equivalent figures for fortified crude sewage (addition of primary sludge accounting for 82% of the tCOD loading), where influent values for E1 varied between 28 and 41 ng 1^−1^, for E2 between 7.5 and 15 ng 1^−1^ for E3 between 47 and 68 ng 1^−1^ and for E1-3S between 33 and 63 ng 1^−1^, that fortification had no significant impact on the influent estrogen load, with the possible exception of E1–3S (Table 2). The removal of estrogens during primary sedimentation is modest,[44] due to their relative high solubility and therefore primary sludge is not a concentrated source of these compounds [47] and given the very small amount added (approximately 7% by volume) this is to be expected.

**Table 2.** Removal of steroid estrogens from fortified (0.8 days HRT), an unfortified crude wastewater (0.2 and 0.4 days HRT) and an anaerobic MBR (0.7 days HRT).

		EGSB unfortified (0.2 days HRT)			EGSB fortified (0.8 days HRT)			EGSB unfortified (0.4 days HRT)				anMBR (0.7 days HRT)		
Compound	Day	Influent	Effluent	% removal	Influent	Effluent	% removal	Day	Influent	Effluent	% removal	Influent	Effluent	% removal
E1 (ng l^−1^)	1	65	19	71	33	13	61	1	41	15	63	41	51	21
	5	36	26	28	28	22	21	5	36	10	72	36	0	100
	10	46	17	63	37	19	49	10	60	28	53	60	7.7	83
	15	16	15	8.5	34	18	47	15	39	16	59	39	7.2	56
	20	7.5	6.9	8.1	41	38	7.3	20	14	3.3	76	14	8.2	−9.2
Average				36%			37%				65%			58%
E2 (ng l^−1^)	1	11	11	0	7.5	2	73	1	22	6	73	22	0.5	95
	5	0.8	0.1	86	10	2	80	5	13	3.5	73	13	0.7	15
	10	4.6	1.7	63	8	3	63	10	17	5.4	68	17	0.9	81
	15	14	8.1	40	12	4	71	15	12	5.1	57	12	0.1	99
	20	2.8	2.8	0	15	4	77	20	9.7	3.3	65	9.7	0.1	96
Average				63%			73%				67%			77%
E3 (ng l^−1^)	1	45	31	31	52	37	29	1	57	31	46	57	28	38
	5	65	11	83	68	24	65	5	61	10	84	61	36	45
	10	53	14	73	47	24	49	10	56	19	65	56	0.9	98
	15	68	26	62	61	58	5	15	42	22	48	42	36	48
	20	60	7.8	87	60	30	50	20	70	21	70	70	17	72
Average				67%			40%				63%			60%
E1-3S (ng l^−1^)	1	43	5	88	62	10	85	1	38	1.3	97	38	0.5	99
	5	30	3	90	63	2	97	5	39	0.4	99	39	0.7	98
	10	41	2.4	94	47	3	94	10	41	3.8	91	41	3.6	91
	15	38	0.9	98	39	11	73	15	30	1.8	94	30	3.3	91
	20	30	1.4	95	33	6	81	20	35	0	100	35	0.8	97
Average				93%			86%				96%			95%

### Impact of HRT on the removal of estrogens in EGSB reactors

It has been suggested that under aerobic conditions HRT is an important factor in determining estrogen removal.[48,19] To determine if this hypothesis was applicable to anaerobic treatment, data from the operation of the EGSB pilot plant at two different HRTs of 4.8 (Table 2) and 9.6 h (Table 2) have been compared without fortification. It is apparent that in general terms increased HRT had a beneficial impact on estrogen removal for E1 from 36% to 65%, E2 from 63% to 67% and for E1–3S from 93% to 96%. This is consistent with process HRT being a significant factor in removal and biodegradation.[48,19] However, for E3, removal was reduced from an average of 67% to 63% at an HRT of 4.8 and 9.6 h, respectively.

In addition, the pilot plant treating crude sewage with and without fortification with primary sludge to achieve constant OLR and an HRT of 4.8 h (OLR of 2.1 kg COD m^3^ d^−1^) and an HRT of 19.4 h (OLR of 2.2 kg COD m^3^ d^−1^) also showed some beneficial improvements in removal at the higher HRT for E1 and E2 (Table 2).

### Impact of fortification on steroid estrogen removal

Two EGSB reactors were operated in parallel one supplied with crude wastewater with an influent of SS 144 mg l^−1^, tCOD 414 mg l^−1^, BOD 247 mg l^−1^ and the other with fortified crude sewage (by the addition of approximately 7% primary sludge by volume) with influent SS of 1431 mg l^−1^, tCOD 1790 mg l^−1^, BOD 1303 mg l^−1^ (Table 1). The reactors were operated for 30 days to allow for stabilization and thereafter the influent and effluent were sampled at 5-day intervals to be analysed for steroid estrogens to facilitate the calculation of percentage removals. These data are presented in Table 1.

Removal of E1 and E2 tended to be slightly higher in the fortified reactor, particularly E2 which was 63% in the unfortified crude sewage reactor, but 73% in the fortified reactor. Removal of E3 was 67% in the unfortified crude sewage reactor which was significantly higher than the 40% achieved in the fortified reactor. A similar but not as pronounced pattern was observed for E1–3S with 93% removal in the unfortified crude sewage reactor and 86% removal in the fortified reactor. This is consistent with the observed behaviour in aerobic systems where estrogen removal has been lower in the presence of high concentrations of easily biodegradable organic substrates. These removals are on average higher than those reported for sludge digestion by [47] under both mesophilic and thermophilic conditions, although those concentrations are somewhat higher in sludges.

The removal of El will be affected by the short residence time (< 3 h) in the sewerage system supplying the pilot plants which meant that the influent contained very high concentrations of E1–3S (30–43 ng 1^−1^ unfortified reactor, 33–63 ng 1^−1^fortified reactor) which would have ordinarily been converted to E1 either in the sewerage network (residence time more typically > 8 h) and in the primary sedimentation tanks (residence time typically 2–6 h). This allows for bacterial sulphonases to convert the sulphated conjugate E1–3S to E1. As can be seen from Table 2 removal of E1–3S in both reactors was very high (>85%), thus providing considerable additional load of E1 to the reactors which may not be able to fully biodegrade this at the lower HRT (0.2 days).

It has been demonstrated [49] that in upflow anaerobic sludge blanket (UASB) reactors treating septic tank waste, sludge was transformed from E1 into E2 but no transformation of E1 occurred in these granular UASB sludge reactors under anaerobic conditions for more than 40 days. Czajka and Londry [50] investigated anaerobic biotransformation of E2 added to the lake sediments under methanogenic-, sulphate-, iron- and nitrate-reducing conditions and demonstrated that E2 was transformed to E1 under all four anaerobic conditions. In this study, E2 was not consistently removed through the anaerobic processes examined as shown in Table 2. However, no significant increases in E2 concentrations in the effluent were observed, even though a high concentration of E1 was present in the effluent.

Furuichi et al. [51] investigated the removal of estrogenic compounds from swine farm wastewater in a treatment process utilizing a UASB reactor at a HRT of 2 days and a trickling filter at a HRT of 1 day and reported that the removal efficiency of E1 (27% and 33%) through the UASB reactor and trickling filter, respectively, was lower than that of E2 (53% and 73%), respectively. Although the concentrations of E1 and E2 in the influent in this study were much lower than those in the swine farm wastewater, the behaviour was consistent with the results of the swine farm wastewater study.

### Comparison of the removal of steroid estrogens in an EGSB and anaerobic MBR

A study was undertaken to compare the influence of reactor type on steroid estrogen removal. The EGSB reactor was operated on unfortified crude sewage with an HRT of 4.8 h in parallel with an anMBR with an HRT of 15.8 h. These retention times were selected to utilize realistic operational regimes of the processes to achieve viable comparisons. The anMBR was observed to have significantly better removal for both E1 (58%) and E2 (77%) (Table 2) due in part to the improved SS removal in this reactor (Table 1) although removal of E1 was consistently lower than anticipated in comparison to biological treatment processes both (aerobic [48,19] and anaerobic [47]). Again this is attributed to the short sewerage retention time at this site and the consequently high E1–3S loading as explained above. This is supported by the high removal/deconjugation of E1–3S in both reactors.

### Performance of fortified EGSB with other anaerobic processes in the removal of organic matter and SS

One of the primary reasons for this study was to establish if anaerobic municipal crude sewage could be treated effectively under temperate conditions, by means of fortification [35] and would effectively remove hazardous substances, for which estrogens were chosen as a model. It has been established not only that estrogens were removed in anaerobic systems such as sewage sludge,[47] but also in less concentrated systems.[49–51] In this study (Table 2) fortification clearly demonstrates the previously established benefits of this route of treatment for BOD removal, with the consequential saving in energy (aeration costs) and sludge production reduction, with consequential benefits for reuse. Given that the effluent from the anaerobic stage can and of necessity needs to be incorporated into the settled sewage stream for subsequent treatment for N and P removal,[41] this route of treatment appears to be highly advantageous.

## Conclusions

1. Concentrations of estrogens in crude sewage in this study were typical of other values reported for influent sewages in the UK and Europe.
2. E2 was readily converted to E1 in all anaerobic configurations.
3. Loadings of individual estrogens appeared to vary with the sewerage system retention time.
4. Biodegradation occurred under anaerobic conditions in both normal strength crude sewage and fortified crude sewage at temperate zone temperatures.
5. Estrogen deconjugation occurred in a manner entirely consistent with that previously observed in other UK Studies.
6. The anaerobic MBR gave the best removal of estrogens, predominantly due to superior solids removal.
